# Feasibility of a High-Dose Inhaled Indomethacin Dry Powder with Dual Deposition for Pulmonary and Oral Delivery

**DOI:** 10.3390/pharmaceutics16101269

**Published:** 2024-09-28

**Authors:** Jamie E. Spahn, Amr Hefnawy, Feng Zhang, Hugh D. C. Smyth

**Affiliations:** Division of Molecular Pharmaceutics and Drug Delivery, College of Pharmacy, The University of Texas at Austin, 2409 University Avenue, Austin, TX 78712, USA; jspahn@utexas.edu (J.E.S.); ahefnawy@utexas.edu (A.H.); feng.zhang@austin.utexas.edu (F.Z.)

**Keywords:** indomethacin, carrier-free, excipient-free, dry powder inhaler, lactose, inhalation aerosol

## Abstract

In this study we have developed a high-dose dry powder inhaler formulation of indomethacin using a novel approach to carrier-based formulations. Specifically, larger drug particles serve as the carrier for the smaller micronized drug particles, such that an inhaled dose is combined with an oral dose. To study this system, the aerosol performance of a standard indomethacin–lactose formulation was compared to carrier-free micronized indomethacin and a drug-as-carrier formulation (a micronized indomethacin–coarse indomethacin blend). Indomethacin with lactose showed a very poor aerosol performance, indicating high adhesion between the drug and carrier. The performance of the carrier-free micronized drug was significantly better, indicating low cohesion. Coarse drug particles as a carrier allowed improved powder flow and aerosol performance while also providing a potential secondary route of absorption of indomethacin, namely oral. An optimal formulation ratio of 1:1 (*w*/*w*) fine indomethacin–coarse indomethacin was developed in this study.

## 1. Introduction

Indomethacin is a nonsteroidal anti-inflammatory drug (NSAID) used in the treatment of acute pain, such as that associated with arthritis [1]. Indomethacin is most commonly administered orally, subcutaneously, or by suppository. While the suppository route is not preferred by patients, the subcutaneous route is not amenable to at-home treatment, and the oral route often leads to GI side effects [2] and treatment discontinuation [3]. Given that the effectiveness of treatment by indomethacin for a wide range of pain conditions is limited by side effects and the slow onset of action [4], the development of an alternative route of administration is imperative. Among the most promising is the delivery of indomethacin by inhalation, which may have the advantage of rapid onset in treating acute pain due to rapid and extensive absorption of small-molecular-weight relatively hydrophobic compounds [5]. 

Inhalation has been used as a route of administration of therapeutics for centuries. Modern devices can be classified by three categories; nebulizers, pressurized metered dose inhalers (pMDIs), and dry powder inhalers (DPIs). Modern dry powder inhalers were first introduced in the late 1960s and have since become an important option for drug development [6]. Some advantages of dry powder inhalers over other routes of inhalation include breath actuation as well as formulation stability. Formulations for DPIs are usually ordered mixtures consisting of larger carrier particles and small micronized drug particles with a typically low drug content (<5%). However, some high dose systems have been developed, and DPIs may be useful in the delivery of low potency therapies for systemic diseases, like pain and headache. 

Inhaled indomethacin has previously been investigated for effects in asthmatic patients [7,8]. Generally, inhaled indomethacin has been well tolerated and no adverse effects have been observed [7,8,9]. Positive effects of inhaled indomethacin were observed, including a reduction in asthma exacerbations in corticosteroids in steroid-dependent asthma. The inhaled dose in this previous study was not reported, although 25 mg was loaded into a nebulizer and administered twice daily, indicating that lung-delivered doses were likely in the low milligram range [7]. Previously, we also studied inhaled indomethacin formulations [10]. We loaded indomethacin using polyelectrolyte complexation into microparticles (approximately 20% drug loading). Around 15 mg of powder was aerosolized from a commercially available device, resulting in approximately 30% respirable fractions. Although acceptable aerosol performance was observed with these composite particles, only a few milligrams of drug were found to be respirable. In all the previous reports for inhaled indomethacin, none have demonstrated doses of more than a few milligrams. Doses for treatment of headache pain in humans are likely to be substantially higher than these previous attempts in developing an inhaled indomethacin. 

Of particular interest is hemicrania continua (HC). HC is an indomethacin-responsive headache disorder and is classified as a trigeminal autonomic cephalalgia (TAC) [11]. Since HC responds to indomethacin, as opposed to other NSAIDs, the intramuscular administration of indomethacin (INDOTEST) has been suggested as a diagnostic test. Subsequently, HC is often treated by the thrice-daily dosing of indomethacin between 25–500 mg/day [12]. The development of inhaled indomethacin via DPI would provide benefit to patients by potentially speeding up the onset of action [13], lowering the dose, and preventing or decreasing GI side effects [14,15]. This advantage may be substantial for patients facing lifelong high-dose treatment and where there are no other treatment options. However, a major challenge for inhaled indomethacin therapy is the high dose requirement. Specifically, a target indomethacin therapeutic plasma range of 0.5–3 microg/mL has been suggested for the treatment of pain associated with headache [16]. Assuming typical absorption for a low-molecular-weight lipophilic drug [5], this equates to a lung-delivered dose of between 3–18 mg. Thus, a high drug loading formulation is required. Fortunately, indomethacin does not suffer from poor taste when dosed orally and, therefore, a high drug loading formulation is feasible [17]. In the case of inhaled indomethacin described in these studies, a 16.7% formulation was prepared for the initial lactose carrier-based formulation. Although this represents a relatively high drug loading compared to typical carrier-based inhaled formulations [18], in the context of indomethacin dosing it represents relatively high excipient loading. The therapeutic dose for inhaled indomethacin is, therefore, governed by the maximum drug and excipient combination that can be dosed to humans. As previously described, the relatively high drug percentage in a DPI formulation may negatively influence powder flow and aerosol performance due to fewer drug–carrier interactions and more drug–drug particle interactions [19]. In the studies described here, we compare traditional carrier-based formulations to carrier-free and a novel coarse drug carrier system for aerosol performance, powder flow, and the potential for simultaneous oral absorption to achieve dose-delivery requirements of indomethacin in hemicrania continua.

## 2. Materials and Methods

### 2.1. Materials

For studies using micronized indomethacin, indomethacin was jet milled according to conditions previously used for a wide range of APIs [20,21,22]. The grinding gas pressure was set at 3.7 bar and the injector gas pressure was set at 4.1 bar. The material was passed through the micronizer once. The terms micronized and milled are used interchangeably in this paper. The inhalation-grade lactose, Lactohale 206, was obtained from DFE Pharma (Klever Strasse 187, Goch, Germany). Magnesium stearate, from a vegetable source, was purchased from Macron Chemicals (Radnor, PA, USA).

### 2.2. Methods

#### 2.2.1. Formulation Preparation

Micronized indomethacin was used as-is for the pure drug-in-capsule formulation. The particle size distribution is described in Table 1. 

##### Lactose Carrier Formulation

For the lactose carrier formulation, a 1:5 (*w*/*w*) micronized indomethacin–lactose with 0.4% magnesium stearate blend was prepared. A batch size of 130 g was pre-blended using a V-blender for 5 min at 15 rpm. The pre-blend was blended using a Leistritz Nano-16 extruder (American Leistritz Extruder Corp., Somerville, NJ, USA) using parameters previously described by our lab [23]. A twin-screw volumetric feeder (Brabender Technologies, Ontario, Canada) was used to control the feed rate at 4 g/min. The screw profile consisted of conveying elements and one GFM mixing element. The screw speed was set at 100 rpm.

To assess blending as a function of processing time, the powder output from the extruder was sampled (n = 3) and the powder was fed back into the extruder for additional mixing cycles (4 runs through the extruder were assessed). Each run was performed at a feed rate of 5.16 g/min.

##### Coarse Drug Carrier Formulation

The coarse drug carrier formulation was prepared as a 1:5 (*w*/*w*) micronized indomethacin–coarse indomethacin blend or 1:1 (*w*/*w*) micronized indomethacin–coarse indomethacin blend. The formulation was blended using a V-blender for 30 min at 34 rpm. The blends were prepared at 1:5 (*w*/*w*) fine to coarse indomethacin and 1:1 (*w*/*w*) fine to coarse indomethacin as a means to help identify the ratio that would lead to proper therapeutic dosing. 

#### 2.2.2. Blend Uniformity

##### Lactose Carrier Formulation—UV–VIS Assay 

An ultraviolet absorbance assay was used to measure blend uniformity for the lactose carrier formulation. A 1 mg/mL indomethacin stock solution was prepared in 100% methanol. The stock was diluted using 90% methanol down to approximately 2 µg/mL, and a range of 250 µg/mL to 2 µg/mL was used as the standard curve. Samples were prepared by measuring 10 mg of powder in 10 mL 90%methanol diluent, then centrifuged at 14,000 rpm for 30 min. A Tecan^®^ Infinite^®^ 200 PRO multimode microplate reader (Tecan Systems, Inc. San Jose, CA, USA) was used with Greiner plates. Ultraviolet measurements were performed at 320 nm.

##### Coarse Drug Carrier Formulation—Particle Size Dispersion (PSD)

For the coarse drug carrier formulation, blend uniformity was measured using a particle size distribution assay to estimate the uniformity of fines distributed throughout the powder. Laser diffraction (HELOS, Sympatec, Am Pulverhaus 1, 38678 Clausthal-Zellerfeld, Germany) was used with RODOS powder dispersion. A pressure drop of 3 bar was used, with the rotor speed set at 50%. Optical concentration measurements between 5% and 25% were included in the analysis. The particle diameter is considered to be the mean Xn diameter values, with n% of the particles having a diameter ≤ X. 

#### 2.2.3. Aerosol Performance

In vitro aerosol performance was measured using a next-generation impactor (NGI) (MSP Corporation, Shoreview, MN, USA). The NGI was attached sequentially to a volumetric digital flow meter (TSI 4000 Series, TSI Performance Measurement Tools, Shoreview, MN, USA), a two-way solenoid valve timer box, and a high-capacity vacuum pump (HCP5, Copley Scientific Limited, Nottingham, UK). A medium-resistance Plastiape (Berry Global, Osnago, Italy) RS01 dry powder inhaler was used with size 3 inhalation grade HPMC Vcaps capsules donated from Capsugel Inc. (Morristown, NJ, USA). To prepare the equipment, 10 mL of 90% methanol was added to the pre-separator, and the stages were coated with a 5 mL solution of 1% (*v*/*v*) of glycerin in ethanol, which was evaporated. The flow rate was determined to be approximately 60 L/min, creating a 4 kPa pressure drop across the device with a total volume of 4 L. A SRH77A thermo-hygrometer by Cooper-Atkins Instrument Corporation (Middlefield, CT, USA) was used to monitor temperature and relative humidity. The capsule, device, and mouthpiece were each washed with 15 mL of 90% methanol. The induction port was washed with 10 mL of 90% ethanol, and the pre-separator was washed with a total of 30 mL 90% ethanol. The stages of the NGI were washed with different volumes of 90% methanol depending on the formulation being tested. Samples were assayed using the UV method described in Section 2.2.2. Lactose Carrier Formulation—UV–VIS Assay. Samples containing lactose were centrifuged at 14,000 rpm for 30 min prior to measurement.

#### 2.2.4. Blend Characterization

##### Particle Size Distribution

Particle size distribution was measured as discussed in Section 2.2.2. Coarse Drug Carrier Formulation—Particle Size Dispersion (PSD).

##### Flow Properties

The angle of repose (AOR) was measured for the coarse and fine indomethacin powders using a Flodex system. Approximately 30 g of powder was weighed and added to the funnel. The powder was allowed to flow through the funnel and onto the stage, forming a cone. The height of the cone was measured, and the angle of repose was determined.

Bulk density and tapped density were measured using a tapped density tester using a 50 mL graduated cylinder and an appropriate volume of powder. The bulk density, compressibility index (CI), and Hausner ratio (HR) were calculated according to USP method <616>.

##### Powder X-ray Diffraction (PXRD)

A Rigaku Miniflex 600 instrument (Rigaku Americas, The Woodlands, TX, USA) equipped with a Cu-Kα radiation source generated at 40 kV and 15 mA was used to perform PXRD. Samples were scanned as a step-wise measurement with a step size of 0.03° over a 2θ range of 4° to 45°.

##### Differential Scanning Calorimetry (DSC)

A DSC Q20 (TA Instruments, New Castle, DE, USA) was used to perform differential scanning calorimetry (DSC). Samples were scanned from 25 °C to 200 °C at a rate of 10 °C/min using approximately 6 mg per sample.

##### Scanning Electron Microscopy (SEM)

Scanning electron microscopy was performed with gold sputter-coated samples on an FEI Quanta 650 instrument.

### 2.3. Statistics

Statistics was implemented using GraphPad Prism version 10.3.1 software. Analysis was performed using t-tests to determine *p*-values.

## 3. Results

### 3.1. Blend Preparation and Uniformity

Indomethacin–lactose (1:5 *w*/*w*) blend and indomethacin–coarse indomethacin (1:5 and 1:1 *w*/*w*) blends were prepared and blend uniformity was measured (Table 2). The indomethacin–lactose blend measured 95.61% with a relative standard deviation (RSD) of 7.6%. The indomethacin–coarse indomethacin blends, because each component was the same chemically, were alternatively measured using particle size distribution analysis to assess uniformity of the coarse fraction and the fine fraction in the blend. The 1:5 (*w*/*w*) fine indomethacin–coarse indomethacin blend had a D_10_ of 1.25 µm and an RSD of 3.0%, and a D_90_ of 37.85 µm with an RSD of 1.8%. The low RSDs indicated high uniformity. The 1:1 (*w*/*w*) fine indomethacin–coarse indomethacin blend also showed good uniformity, with a D_10_ of 0.86 µm and a D_90_ of 22.81 µm with an RSD of 3.5%. The low value for D_10_ (1.25 µm for the 1:5 coarse drug carrier formulation) indicates that a large portion of the particles in the formulation are potentially respirable. 

### 3.2. Powder Characterization

#### 3.2.1. Particle Size and Morphology (Particle Size Distribution (PSD) and Scanning Electron Microscopy (SEM))

**Figure 1:**
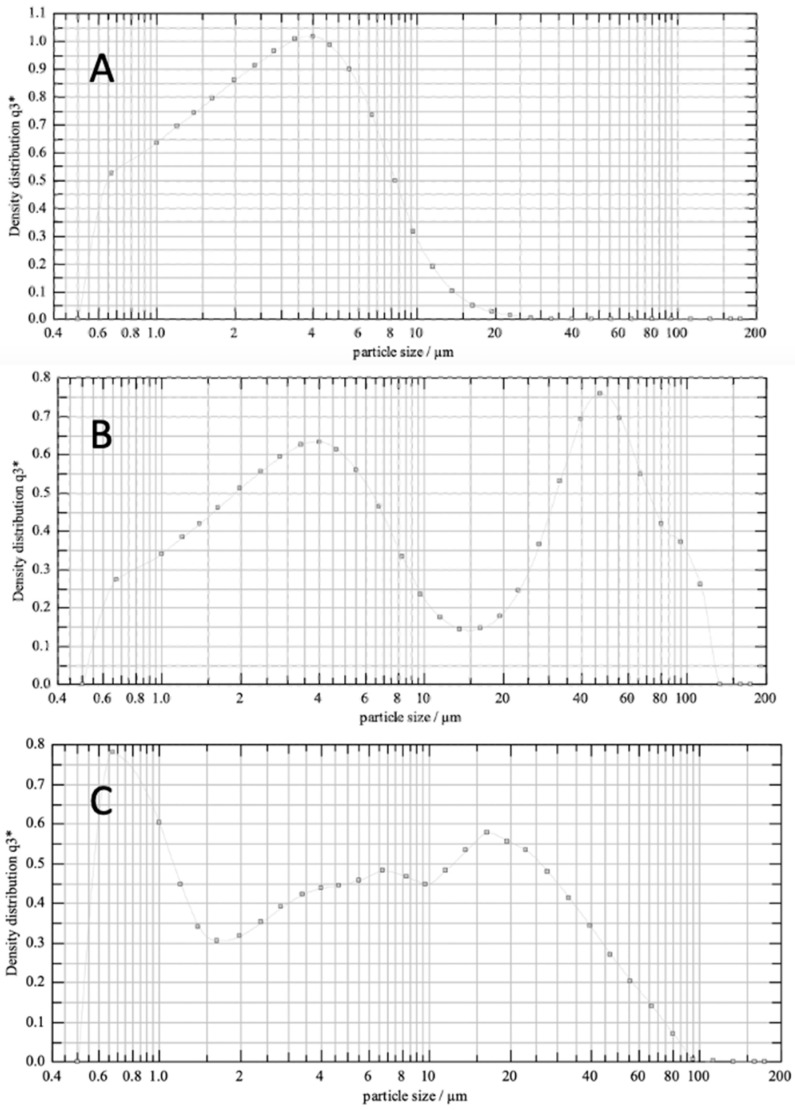
Particle size distribution. (**A**) Micronized indomethacin. (**B**) 1:5 (*w*/*w*) micronized indomethacin–lactose. (**C**) 1:5 (*w*/*w*) micronized indomethacin–coarse indomethacin.

**Figure 2:**
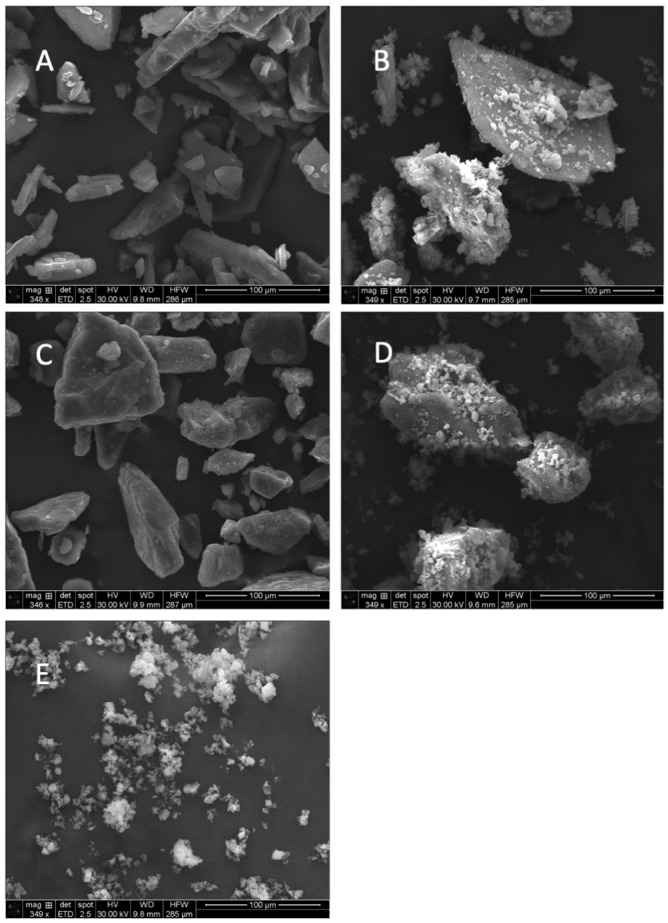
Scanning electron microscopy. (**A**) Coarse indomethacin. (**B**) 1:5 (*w*/*w*) micronized–coarse indomethacin blend. (**C**) Lactose with 0.4% (*w*/*w*) magnesium stearate. (**D**) 1:5 (*w*/*w*) micronized indomethacin–lactose blend. (**E**) Micronized indomethacin.

Particle size distributions (Figure 1 and Table 1) showed a single peak for micronized indomethacin and a bimodal distribution for the indomethacin–lactose formulation and the fine indomethacin–coarse indomethacin formulation. Regarding the carrier particles, lactose was demonstrated to be larger in size (D_90_ of 116.1 µm) as compared to coarse indomethacin (D_90_ of 42.5 µm). Scanning electron microscopy revealed a difference in the particle morphology (Figure 2). Lactose carrier particles were, as expected, tomahawk-like structures [24], while coarse indomethacin were plate-like structures [25]. Upon the addition of micronized indomethacin, both blends showed significant coating of the micronized indomethacin on the respective carrier (Figure 2A and Figure 2B, Figure 2C and Figure 2D, respectively).

#### 3.2.2. Powder Physicochemical Properties (PXRD and DSC)

**Figure A1:**
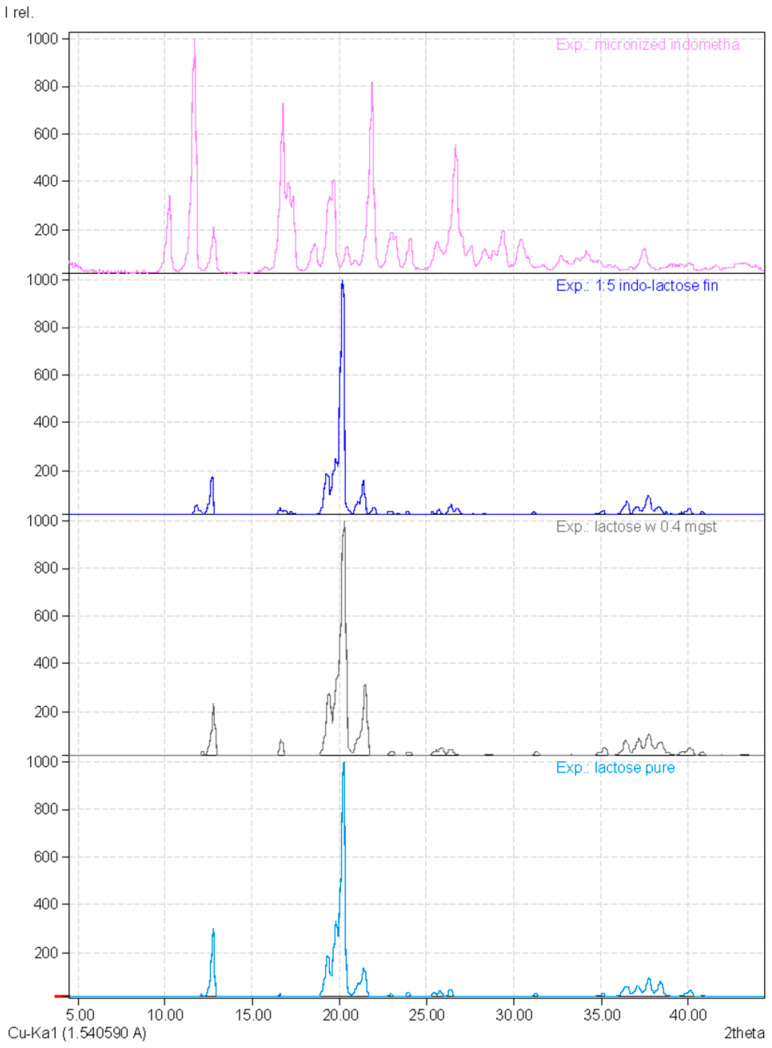
Powder X-Ray diffraction (PXRD). Blends described are formulated as (*w*/*w*).

**Figure A2:**
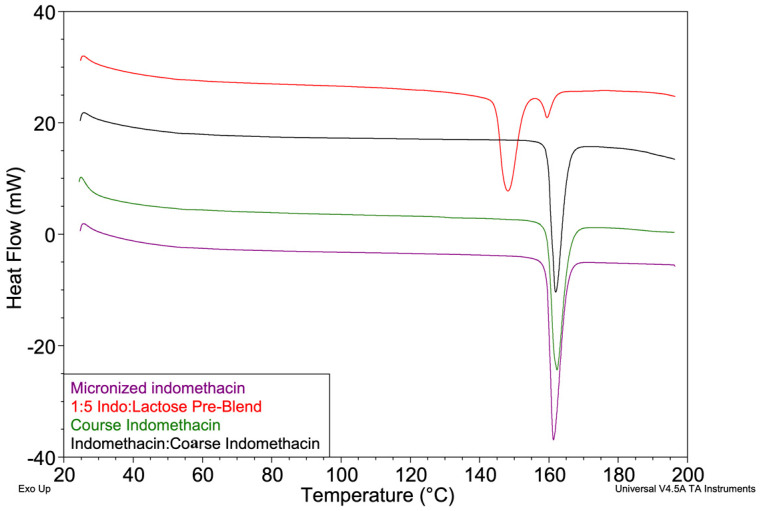
Differential scanning calorimetry (DSC). Blends described are formulated as (*w*/*w*).

The physicochemical properties of the powder blends were assessed using PXRD and DSC (Appendix A Figure A1 and Figure A2). PXRD showed no change in the crystalline structure in the indomethacin–lactose blend upon blending with fine indomethacin. DSC confirmed this finding, displaying the gamma crystalline form of indomethacin for all blends, while showing an additional lactose peak for the lactose blend. It has previously been reported that amorphous indomethacin undergoes rapid surface crystallization [26] which would account for any surface amorphous content introduced by air jet milling.

#### 3.2.3. Powder Flow Properties

Powder flow was measured in triplicate using the angle of repose (AOR) for coarse and fine indomethacin. The AOR for milled indomethacin was 46.73 ± 3.00, which is designated as poor flow, which must be agitated or vibrated. The AOR for coarse indomethacin was slightly better at 38.99 ± 0.58 with a designation of fair flow, and aid not needed [27]. 

The bulk density and tapped density of coarse and milled indomethacin were measured according to USP <616>. The results are shown in Table 3.

#### 3.2.4. Powder Interparticulate Properties

**Figure 3:**
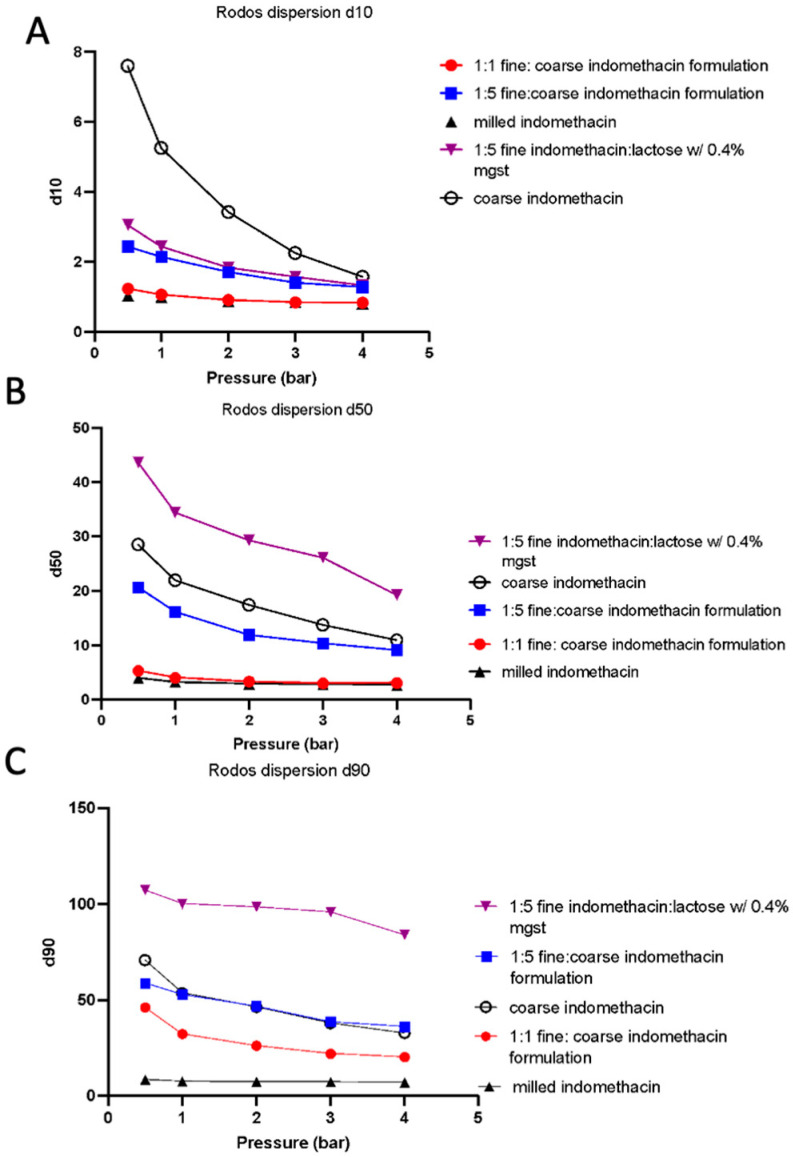
RODOS dispersion of indomethacin formulations. (**A**) D_10_, (**B**) D_50_, (**C**) and D_90_. Blends described are formulated as (*w*/*w*).

A RODOS dispersion study was performed at 0.5, 1, 2, 3, and 4 bar to establish the changes in particle size as a function of dispersion pressure that introduces the powder to the laser diffraction instrument. The results can be seen in Figure 3, where 1:5 (*w*/*w*) fine indomethacin–lactose, coarse indomethacin, and 1:5 (*w*/*w*) fine indomethacin–coarse indomethacin all show a downward slope, indicating that full deagglomeration has not been achieved at the tested dispersion pressures. Alternatively, 1:1 (*w*/*w*) fine indomethacin–coarse indomethacin and milled indomethacin both show flat profiles, indicating that beyond a certain point, deaggregation is independent of the shear force applied. Therefore, these formulations show lower interparticulate forces that often correlate with improved aerosol performance.

Rodos dispersion curves of the indomethacin and lactose formulations reveal interesting dispersion pressure dependencies of particle redispersion. The milled indomethacin powders displayed relatively constant particle sizes regardless of dispersion pressure. In contrast, both lactose blends and the coarse indomethacin powder particle sizes showed significant inverse proportionality with dispersion pressures, indicating that these powders require significantly more energy to disperse indomethacin as an aerosol. Blends of fine and coarse indomethacin show an intermediate dispersion dependency on dispersion pressures with the 1:1 blend, resembling the pure micronized material.

### 3.3. Aerosol Performance Results

A summary of the aerosol performance of each formulation and capsule loading is presented in Table 4. A lactose blend formulation showed poor performance, with only 0.3 mg out of the loaded 5 mg being classified in the fine particle range (and a subsequent fine particle fraction of only around 7%). In contrast, pure micronized and blends of micronized indomethacin with coarse indomethacin resulted in fine particle doses greater than 1 mg for a 5 mg loading. Expectedly, in the indomethacin-only formulations, as the amount of micronized drug was increased, the fine particle dose also increased. Generally, the emitted fractions of the 1:1 (*w*/*w*) micronized–coarse blends were higher than the pure micronized powders (for highest fill, *p* = 0.02) 

**Figure 4:**
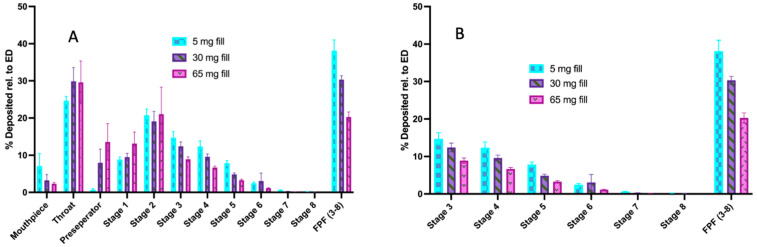
Fine particle fraction of pure micronized indomethacin at increasing fill weights. (**A**) Total aerosol performance. (**B**) Aerosol performance on stages 3–8.

Figure 4 shows the influence of the drug mass loaded into capsules on the resulting stage deposition using the next-generation impactor for the pure micronized indomethacin powder. For this powder, as loading was increased, the fine particle fraction decreased (although the overall fine particle dose increased, see for example Table 4). This was driven by the increased drug deposition on the upper stages (pre-separator through stage 2) and the subsequent lower deposition on stages 3–7. 

**Figure 5:**
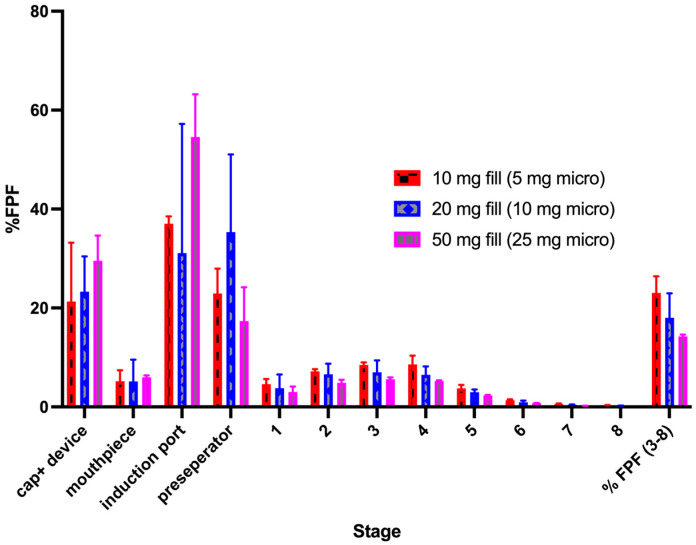
Aerosol performance of the 1:1 (*w*/*w*) micronized indomethacin–coarse indomethacin formulation.

The aerosol deposition of the 1:1 micronized to coarse indomethacin formulation is shown in Figure 5. Similar to pure micronized indomethacin, as the powder loading was increased, the fine particle fraction decreased, although the differences were not as pronounced. Despite the general decreases in efficiency, as quantified by FPF, the fine particle doses for the highest powder loadings of the pure micronized drug (65 mg) and the 1:1 (*w*/*w*) blends (50 mg) were found to have fine particle doses above 3 mg. 

**Figure 6:**
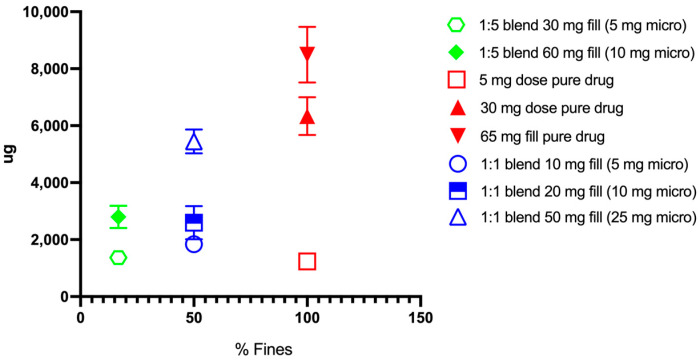
Fine particle dose as a function of percent fines (*w*/*w*) for indomethacin-only formulations. Blends described are formulated as (*w*/*w*).

Despite the general decreases in efficiency, as quantified by FPF, the fine particle doses of the pure micronized drug and the 1:1 blends were found to have FPDs above 3 mg (see discussion below), as shown in Figure 6. 

## 4. Discussion

In addition to a lactose carrier formulation, the aerosol performance of two other indomethacin formulations was compared. First, micronized indomethacin without a carrier was studied, and this was observed to have superior performance. Additionally, a novel formulation method was developed using coarse indomethacin particles as the fine particle carrier. This system also showed superior aerosol performance compared to the lactose carrier formulation. An additional benefit to the coarse drug carrier formulation includes the potential for a secondary route of administration, namely oral. Literature suggests a therapeutic range of 0.5–3 microgram/mL for indomethacin [16]. Past work shows that 25 mg indomethacin IV achieves a peak plasma concentration of 4 microgram/mL [28]. Assuming the fine particle dose of indomethacin will be absorbed similar to the intravenous (IV) dose, if we target a 0.5–3 microgram/mL plasma concentration, an approximate inhaled fine particle dose will be approximately 3–18 mg. Since the 1:5 (*w*/*w*) formulation achieved an approximately 2 mg fine particle dose, we determined the optimal ratio to be 1:1 (*w*/*w*) to achieve a fine particle dose within the specified range. This drug-as-carrier system for an indomethacin DPI was, therefore, further studied. This system shows promise for alternative drug candidates sharing similar physicochemical properties that have a high dose requirement. 

### 4.1. Lactose Blend Formulations Had Poorer Aerosol Performance Compared to Indomethacin-Only Formulations

Typical dry powder inhalation formulations have revolved around the concept of adhesive or interactive mixtures of the micronized drug with inert carrier particles [29]. The general working principle with these carrier-based systems is that the inert carrier particle (most frequently lactose alpha monohydrate) facilitates improvements in powder flow and aerosol performance [30]. This function relies upon the interactions between the drug and carrier particles being sufficient such that the drug adheres to the carrier particle to enable homogenous blending, but that the interaction is also sufficiently weak such that the drug particles can be detached during inhalation to allow for lung deposition [31]. Begat et al. [32] described the concept of the cohesive–adhesive balance of different model dry powder inhaler systems, showing that different APIs had different interactions with lactose. In the studies presented here, we show that indomethacin–lactose interactions appear to be greater than indomethacin–indomethacin interactions, as indicated by both powder interparticulate studies (Rodos dispersion) and the lower fine particle doses achieved with lactose formulations during aerosol performance testing. Specifically, for the 5 mg micronized indomethacin formulations, the lactose formulation fine particle doses averaged only 0.48 mg compared to 1.3 mg and 1.2 mg for the coarse drug and carrier free indomethacin formulations, respectively. The Rodos dispersion studies show the micronized indomethacin material had less dispersion pressure dependency than carrier-based (either lactose or coarse indomethacin). Collectively, these aerosol performance and powder dispersion studies indicate that indomethacin–lactose carrier formulations are more difficult to disperse, though it is not clear if this is a result of differences in the adhesion of the indomethacin with lactose and the cohesion between indomethacin particles. 

### 4.2. Indomethacin-Only Formulations Achieve Target Respirable Doses 

Indomethacin has been studied for the treatment of pain for decades, and the pharmacodynamic relationship between plasma concentrations and efficacy has been well established [33]. Specifically, a target indomethacin therapeutic plasma range of 0.5–3 microg/mL has been determined for the treatment of pain associated with headache [16]. In this study, we used this as a guide for developing target doses for inhaled indomethacin. Because indomethacin is relatively lipophilic and small in molecular weight it is assumed that pulmonary absorption would be rapid and extensive, similar to that of parenteral administration [12]. Previous studies of intravenously administered indomethacin showed that 25 mg indomethacin achieves a peak plasma concentration of 4 micrograms/mL [28]. Therefore, targeting a 0.5–3 microgram/mL plasma concentration for the rapidly absorbed pulmonary fraction of inhaled indomethacin, we estimated that a fine particle dose of between 3–18 mg would be required for the acute treatment of headache pain. Thus, in these studies we investigated the influence of the mass of micronized indomethacin and the effects of the ratio of coarse to fine indomethacin on the ability to achieve FPDs on the order 3–18 mg. We found that as the total fill was increased in formulations with high proportions of micronized indomethacin, and that decreased fine particle fractions were observed (Figure 4). The ratios used in this study (1:1 and 1:5 fine to coarse indomethacin) may ultimately not be ideal, and further optimization of the ratio is warranted. 

However, for the formulations with a higher content of coarse indomethacin (i.e., 1:5 (*w*/*w*) blends) aerosol performance increased with increased fill, albeit with increased variability for FPF (Figure 6). The variability in the lead formulations in our studies (i.e., the 1:1 (*w*/*w*) micronized to coarse indomethacin), as detailed in Table 4, was found to be at an acceptable level and within typical limits for inhaled products. For example, the optimal formulation to achieve adequate dosing (as described above) was the 1:1 (*w*/*w)* micronized to coarse formulation with a high loaded mass. The variability in the emitted dose, as measured by the percent coefficient of variation (%CV), was less than 6%. The variability in the fine particle dose was 7.7%, and the variability in the coarse particle fraction as outlined in Table 5 was 6%. Compared to lactose formulation (i.e., %CV on the order of 18%, Figure 5) these demonstrated low variability and are promising for future development.

### 4.3. A Coarse Particle Dose, When Using Indomethacin-Only Formulations, Is Likely to Provide Adequate Oral Dose for Additional Oral Absorption and Prolonged Plasma Levels 

Indomethacin given orally has high bioavailability [28]. The prescribing information for indomethacin capsules, for example, indicates that the bioavailability is approximately 100% and that around 90% of the dose is absorbed within 4 h of dosing [34]. Despite this high bioavailability, the time to peak plasma concentrations is relatively delayed compared to the clinical need to treat acute headache. In the fasting state, Tmax occurs between 0.9 and 1.5 h [28,35]. Thus, the rapid absorption of micronized indomethacin from the airways has the potential to bridge the gap in therapeutic levels observed upon oral administration of indomethacin. The indomethacin-only formulations studied here not only provide the potential for rapid onset of lung-delivered drug, but also provide this oral dose. As shown in Table 5, the coarse particle dose (CPD) for both the micronized-only and coarse–fine indomethacin formulations also provide a significant dose that will be deposited in the oropharyngeal cavity and swallowed. For the micronized indomethacin-only formulation, this oral dose is estimated to be 14.64 mg for a 30 mg fill (Table 5), while for the 1:1 coarse indomethacin blend this oral dose is 12.19 mg for a 20 mg fill (Table 5). Assuming the oral pharmacokinetics remain the same for these powders, adequate plasma levels should be attained at prolonged time periods after the initial phase of absorption from the pulmonary compartment has occurred.

### 4.4. Coarse Indomethacin Demonstrates Superior Flow over Milled Indomethacin

Using the angle of repose, coarse indomethacin demonstrated superior flow properties over milled indomethacin (*p* = 0.012). This indicates that the coarse–fine indomethacin blend would also have superior flow properties over the micronized indomethacin, suggesting a benefit for the novel formulation over the carrier-free formulation during processing.

Powder flow analyses of the micronized and coarse indomethacin powders revealed interesting observations that can be related to both powder filling and aerosol performance. Firstly, it can be seen from the angle of repose (AOR) data that coarse indomethacin has improved flow (Table 3). Significant differences were observed with the coarse AOR corresponding to a “fair” flowability, while the micronized was classified in the “poor” category. For capsule filling operations using commercial filling equipment, variability is correlated with AOR, Carr’s index, and Hausner’s ratio [36]. Thus, it can be expected that capsule fill variations would be minimized using the coarse indomethacin blends.

## 5. Conclusions

A novel formulation was developed using a coarse drug carrier for the high dose dry powder inhaled indomethacin. This formulation consists of micronized drug particles adhered to larger, coarse drug particles. This micronized drug–coarse drug blend, in comparison to the standard micronized drug–lactose blend, allows an inhaled dose and a secondary oral dose to be administered in addition to the inhaled dose. In terms of aerosol performance, carrier-free and coarse drug carrier formulations showed similar performance, indicating the addition of the coarse drug carrier did not compromise the superior aerosol performance relative to the micronized drug–lactose blend. However, improvements in the powder flow were observed for the coarse–drug blends. A 1:1 (*w*/*w*) formulation of micronized–coarse indomethacin was determined to be the optimal blend for depositing a relevant lung and oral dose for the treatment of hemicrania continua.

Inhaled therapeutics have been used beyond applications in asthma and COPD and include diseases, such as rescue medication in Parkinson’s disease and rapidly acting insulin to name a few. For hemicrania continua we anticipate a high degree of patient acceptability with this modality of treatment. The rapid onset and prolonged action obtained through the dual routes of absorption would be especially attractive. As an analogy, intranasal zolmitriptan, a standard commercialized treatment for migraine, also has an observed biphasic pharmacokinetic profile (rapid onset within minutes from nasal absorption and second-phase absorption through a swallowed fraction) which is marketed as a significant clinical benefit for the onset and duration of action. Lastly, the clinical experience of oral cavity-administered indomethacin also provides evidence of suitability in terms of taste [17].

## Figures and Tables

**Table 1:** Particle size distribution of indomethacin formulations.

Formulation	X_10_	X_50_	X_90_	Span ((X_90_ − X_10_)/X_50_)
Lactohale 206	1.9 µm	4.2 µm	116.1 µm	27.5
Lactohale 206, 0.4% magnesium stearate	5.3 µm	54.8 µm	101.4 µm	1.8
Micronized indomethacin	0.8 µm	2.7 µm	7.3 µm	2.4
Coarse indomethacin	2.6 µm	15.7 µm	42.5 µm	2.5
1:5 (*w*/*w*) micronized indomethacin–lactose	1.2 µm	11.7 µm	64.1 µm	5.4
1:5 (*w*/*w*) micronized indomethacin–coarse indomethacin	1.3 µm	10.1 µm	37.9 µm	3.62

**Table 2:** Blend uniformity for lactose and coarse carrier formulations. Sample size n = 3.

			Average % Recovered	RSD (%)
Lactose carrier formulation			95.6	7.6
	**Average d_10_**	**RSD (%)**	**Average D_90_**	**RSD (%)**
1:5 (*w*/*w*) Coarse drug carrier formulation	1.3 µm	3.0	37.9 µm	1.8
1:1 (*w*/*w*) Coarse drug carrier formulation	0.9 µm	2.0	22.8 µm	3.5

**Table 3:** Bulk density and tapped density measurements for coarse and milled indomethacin (n = 3, mean ± standard deviation).

Powder	Angle of Repose (°)	Bulk Density (g/mL)	Tapped Density (g/mL)	Compressibility Index	Hausner Ratio
Coarse indomethacin	38.99 ± 0.58	0.380 ± 0.01	0.61 ± 0.01	37.7 ± 1.2	1.6 ± 0.0
Milled indomethacin	46.73 ± 3.00	0.217 ± 0.00	0.35 ± 0.00	38.9 ± 0.0	1.6 ± 0.0

**Table 4:** Summary of aerosol performance of indomethacin formulations.

Formulation	Micronized Drug Mass (mg)	Carrier Mass (mg)	Emitted Dose (mg)	SD	Fine Particle Dose (mg)	SD	Emitted Fraction (%)	SD	Fine Particle Fraction (%)	SD
Lactose	5	25	3.86	0.66	0.33	0.02	77.2	13.3	6.6	0.4
Pure micronized	5	0	3.26	0.51	1.23	0.12	65.2	10.2	24.6	2.4
	30	0	20.98	2.86	6.34	0.67	69.9	9.5	21.1	2.2
	65	0	41.80	2.24	8.49	0.98	64.3	3.5	13.1	1.5
Micronized + coarse (1:1 *w*/*w*)	5	5	8.12	1.55	1.84	0.12	81.2	15.5	36.7	2.3
	10	10	14.79	2.74	2.60	0.58	73.9	13.7	26.0	5.8
	25	25	38.26	2.23	5.45	0.42	76.5	4.5	21.8	1.7
Micronized + coarse (1:5 *w*/*w*)	5	25	25.74	2.90	1.37	0.17	85.8	9.7	27.4	3.4
	10	50	28.27	16.80	2.80	0.39	47.1	28.0	28.0	3.9

**Table 5:** Coarse particle fraction of indomethacin formulations determined as the dose deposited in the mouth and lungs (=emitted dose—dose deposited in stages 3–8).

Formulation	Micronized Drug Mass (mg)	Carrier Mass (mg)	Coarse Particle Dose (ug)	SD	%CV
Lactose	5	25	3530	641	18
Pure micronized	5	0	2026	400	20
	30	0	14,640	2201	15
	65	0	33,307	1375	4
Micronized + coarse (1:1 *w*/*w*)	5	5	6280	1444	23
	10	10	12,191	2866	24
	25	25	32,813	1830	6
Micronized + coarse (1:5 *w*/*w*)	5	25	24,371	2807	12
	10	50	22,688	17,538	77

## Data Availability

The data presented in this study are available on request from the corresponding author due to activities related to the commercialization of research findings.

## References

[1] O’Brien M., McCauley J., Cohen E., Florey K. (1984). Indomethacin. Analytical Profiles of Drug Substances.

[2] Boardman P.L., Hart F.D. (1967). Side-effects of indomethacin. Ann. Rheum. Dis..

[3] Prakash S., Husain M., Sureka D.S., Shah N.P., Shah N.D. (2009). Is there need to search for alternatives to indomethacin for hemicrania continua? Case reports and a review. J. Neurol. Sci..

[4] Nalamachu S., Wortmann R. (2014). Role of Indomethacin in Acute Pain and Inflammation Management: A Review of the Literature. Postgrad. Med..

[5] Patton J.S., Byron P.R. (2007). Inhaling medicines: Delivering drugs to the body through the lungs. Nat. Rev. Drug Discov..

[6] Bell J.H., Hartley P.S., Cox J.S.G. (1971). Dry powder aerosols I: A new powder inhalation device. J. Pharm. Sci..

[7] Tamaoki J., Nakata J., Nishimura K., Kondo M., Aoshiba K., Kawatani K., Nagai A. (2000). Effect of inhaled indomethacin in asthmatic patients taking high doses of inhaled corticosteroids. J. Allergy Clin. Immunol..

[8] Shimizu T., Mochizuki H., Shigeta M., Morikawa A. (1997). Effect of inhaled indomethacin on exercise-induced bronchoconstriction in children with asthma. Am. J. Respir. Crit. Care Med..

[9] Onischuk A.A., Tolstikova T.G., Sorokina I.V., Zhukova N.A., Baklanov A.M., Karasev V.V., Dultseva G.G., Boldyrev V.V., Fomin V.M. (2008). Anti-inflammatory Effect from Indomethacin Nanoparticles Inhaled by Male Mice. J. Aerosol Med. Pulm. Drug Deliv..

[10] Ceschan N.E., Bucalá V., Mateos M.V., Smyth H.D.C., Ramírez-Rigo M.V. (2018). Carrier free indomethacin microparticles for dry powder inhalation. Int. J. Pharm..

[11] Dodick D.W. (2004). Indomethacin-responsive headache syndromes. Curr. Pain Headache Rep..

[12] Prakash S., Patel P. (2017). Hemicrania continua: Clinical review, diagnosis and management. J. Pain Res..

[13] Cingolani E., Alqahtani S., Sadler R.C., Prime D., Stolnik S., Bosquillon C. (2019). In vitro investigation on the impact of airway mucus on drug dissolution and absorption at the air-epithelium interface in the lungs. Eur. J. Pharm. Biopharm..

[14] Smyth H.D.C., Saleem I., Donovan M., Verschraegen C.F. (2008). Pulmonary Delivery of Anti-Cancer Agents. Advanced Drug Formulation Design to Optimize Therapeutic Outcomes.

[15] Sheikh Z., Ong H.X., Pozzoli M., Young P.M., Traini D. (2018). Is there a role for inhaled anti-inflammatory drugs in cystic fibrosis treatment?. Expert Opin. Orphan Drugs.

[16] Lucas S. (2016). The Pharmacology of Indomethacin. Headache J. Head Face Pain.

[17] Nagaoka H., Momo K., Hamano J., Miyaji T., Oyamada S., Kawaguchi T., Homma M., Yamaguchi T., Morita T., Kizawa Y. (2021). Effects of an Indomethacin Oral Spray on Pain Due to Oral Mucositis in Cancer Patients Treated with Radiotherapy and Chemotherapy: A Double-Blind, Randomized, Placebo-Controlled Trial (JORTC-PAL04). J. Pain Symptom Manag..

[18] Hebbink G.A., Jaspers M., Peters H.J.W., Dickhoff B.H.J. (2022). Recent developments in lactose blend formulations for carrier-based dry powder inhalation. Adv. Drug Deliv. Rev..

[19] Brunaugh A.D., Smyth H.D.C. (2018). Formulation techniques for high dose dry powders. Int. J. Pharm..

[20] Yazdi A.K., Smyth H.D.C. (2017). Implementation of design of experiments approach for the micronization of a drug with a high brittle–ductile transition particle diameter. Drug Dev. Ind. Pharm..

[21] Yazdi A.K., Smyth H.D.C. (2016). Carrier-free high-dose dry powder inhaler formulation of ibuprofen: Physicochemical characterization and in vitro aerodynamic performance. Int. J. Pharm..

[22] Brunaugh A.D., Jan S.U., Ferrati S., Smyth H.D.C. (2017). Excipient-Free Pulmonary Delivery and Macrophage Targeting of Clofazimine via Air Jet Micronization. Mol. Pharm..

[23] Spahn J.E., Hefnawy A., Smyth H.D.C., Zhang F. (2022). Development of a novel method for the continuous blending of carrier-based dry powders for inhalation using a co-rotating twin-screw extruder. Int. J. Pharm..

[24] Clydesdale G., Roberts K.J., Telfer G.B., Grant D.J.W. (1997). Modeling the Crystal Morphology of *α*-lactose Monohydrate. J. Pharm. Sci..

[25] Slavin P.A., Sheen D.B., Shepherd E.E.A., Sherwood J.N., Feeder N., Docherty R., Milojevic S. (2002). Morphological evaluation of the γ-polymorph of indomethacin. J. Cryst. Growth.

[26] Wu T., Yu L. (2006). Surface Crystallization of Indomethacin Below Tg. Pharm. Res..

[27] The United States Pharmacopeia (2016). <1174>Powder Flow [Internet]. https://www.usp.org/sites/default/files/usp/document/harmonization/gen-chapter/g05_pf_30_6_2004.pdf.

[28] Alván G., Orme M., Bertilsson L., Ekstrand R., Palmér L. (1975). Pharmacokinetics of indomethacin. Clin. Pharmacol. Ther..

[29] Grasmeijer F., Grasmeijer N., Hagedoorn P., Frijlink H.W., Haaije de Boer A. (2015). Recent advances in the fundamental understanding of adhesive mixtures for inhalation. Curr. Pharm. Des..

[30] Donovan M.J., Smyth H.D.C. (2010). Influence of size and surface roughness of large lactose carrier particles in dry powder inhaler formulations. Int. J. Pharm..

[31] Spahn J.E., Zhang F., Smyth H.D.C. (2022). Mixing of dry powders for inhalation: A review. Int. J. Pharm..

[32] Begat P., Morton D.A.V., Staniforth J.N., Price R. (2004). The Cohesive-Adhesive Balances in Dry Powder Inhaler Formulations I: Direct Quantification by Atomic Force Microscopy. Pharm. Res..

[33] Yeh K.C. (1985). Pharmacokinetic overview of indomethacin and sustained-release indomethacin. Am. J. Med..

[34] Food and Drug Administration (2019). INDOCIN (Indomethacin) Capsules, for Oral Use [Internet]. https://www.accessdata.fda.gov/drugsatfda_docs/label/2019/016059s100lbl.pdf.

[35] Turakka H., Airaksinen M.M. (1974). Biopharmaceutical assessment of phenylbutazone and indomethacin preparations. Ann. Clin. Res..

[36] Tan S.B., Newton J.M. (1990). Powder flowability as an indication of capsule filling performance. Int. J. Pharm..

